# Genomic mutational analysis of the impact of the classical strain improvement program on β–lactam producing *Penicillium chrysogenum*

**DOI:** 10.1186/s12864-015-2154-4

**Published:** 2015-11-14

**Authors:** Oleksandr V. Salo, Marco Ries, Marnix H. Medema, Peter P. Lankhorst, Rob J. Vreeken, Roel A. L. Bovenberg, Arnold J. M. Driessen

**Affiliations:** Molecular Microbiology, Groningen Biomolecular Sciences and Biotechnology Institute, University of Groningen, Groningen, The Netherlands; Division of Analytical Biosciences, Leiden/Amsterdam Center for Drug Research, Leiden, The Netherlands; Netherlands Metabolomics Centre, Leiden University, Leiden, The Netherlands; Bioinformatics, Wageningen University, Wageningen, The Netherlands; DSM Biotechnology Centre, Delft, The Netherlands; Synthetic Biology and Cell Engineering, Groningen Biomolecular Sciences and Biotechnology Institute, University of Groningen, Groningen, The Netherlands; Kluyver Centre for Genomics of Industrial Fermentations, Julianalaan 67, 2628BC Delft, The Netherlands

**Keywords:** Penicillium chrysogenum, Secondary metabolism, Sorbicillinoids, Polyketide synthases, Sequencing

## Abstract

**Background:**

*Penicillium chrysogenum* is a filamentous fungus that is employed as an industrial producer of β–lactams. The high β–lactam titers of current strains is the result of a classical strain improvement program (CSI) starting with a wild-type like strain more than six decades ago. This involved extensive mutagenesis and strain selection for improved β–lactam titers and growth characteristics. However, the impact of the CSI on the secondary metabolism in general remains unknown.

**Results:**

To examine the impact of CSI on secondary metabolism, a comparative genomic analysis of β-lactam producing strains was carried out by genome sequencing of three *P. chrysogenum* strains that are part of a lineage of the CSI, i.e., strains NRRL1951, Wisconsin 54-1255, DS17690, and the derived penicillin biosynthesis cluster free strain DS68530. CSI has resulted in a wide spread of mutations, that statistically did not result in an over- or underrepresentation of specific gene classes. However, in this set of mutations, 8 out of 31 secondary metabolite genes (20 polyketide synthases and 11 non-ribosomal peptide synthetases) were targeted with a corresponding and progressive loss in the production of a range of secondary metabolites unrelated to β–lactam production. Additionally, key Velvet complex proteins (LeaA and VelA) involved in global regulation of secondary metabolism have been repeatedly targeted for mutagenesis during CSI. Using comparative metabolic profiling, the polyketide synthetase gene cluster was identified that is responsible for sorbicillinoid biosynthesis, a group of yellow-colored metabolites that are abundantly produced by early production strains of *P. chrysogenum.*

**Conclusions:**

The classical industrial strain improvement of *P. chrysogenum* has had a broad mutagenic impact on metabolism and has resulted in silencing of specific secondary metabolite genes with the concomitant diversion of metabolism towards the production of β–lactams.

**Electronic supplementary material:**

The online version of this article (doi:10.1186/s12864-015-2154-4) contains supplementary material, which is available to authorized users.

## Background

**Fig. 1:**
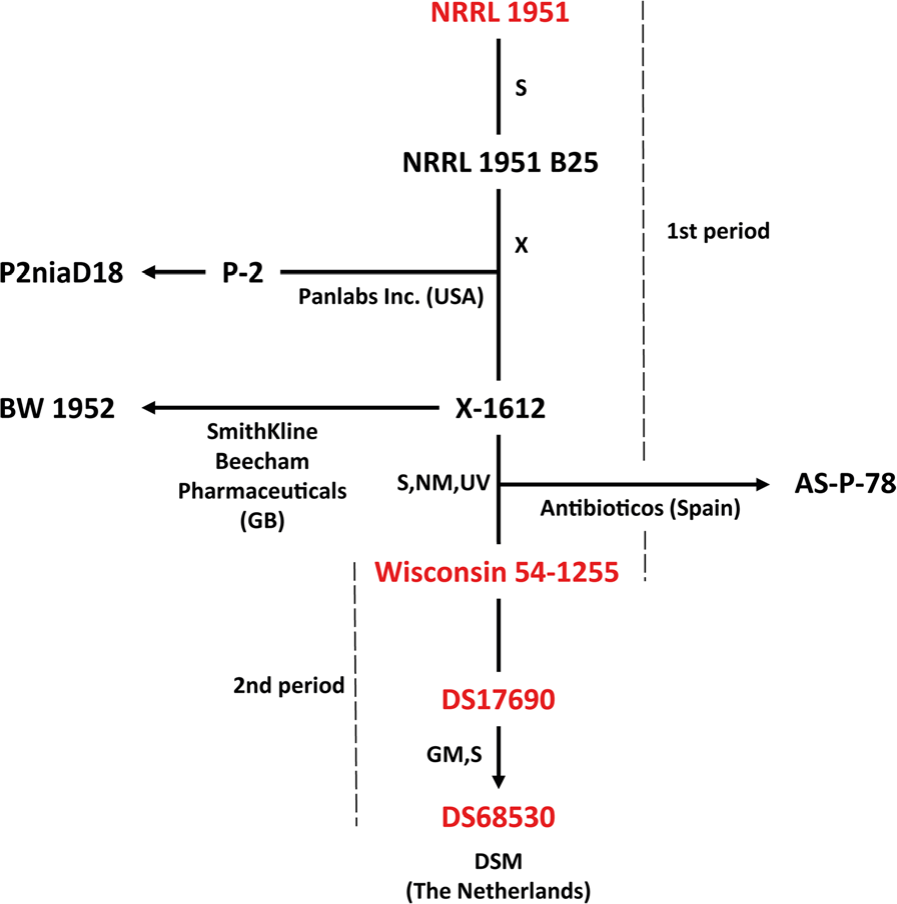
Schematic representation of main industrial β-lactam production improvement programs of *P. chrysogenum.* The main lineages of strains subjected to industrial β-lactam production classical strain improvement programs are shown. Strains NRRL1951 (4 mg/gDW), Wisconsin 54-1255 (20 mg/gDW), and DS17690 (95 mg/gDW) examined in this study are shown in red. In brackets the production levels of penicillin V are indicated according to reference [31]. The penicillin cluster free strain DS68530 that no longer produces penicillins is not included but it is derived from strain DS17690 by genetically removing the multiple penicillin biosynthetic gene clusters [28]. When reported, the techniques used for mutagenesis are indicated according to Newbert et al. [8]. Used abbreviations: S, selection of the spontaneous variant; X, X-Ray irradiation; UV, ultraviolet irradiation; NM, nitrogen mustard (methyl-bis(β-chloroethyl) amine) treatment; MG, other molecular genetic manipulations. Also indicated are the periods 1 and 2 followed in this study in order to identify major steps in the CSI

For the industrial production of β–lactams, the filamentous fungus *Penicillium chrysogenum* has been subjected to an intense classical strain improvement (CSI) program that consisted of repeating rounds of mutagenesis and selection. This program started in 1943 with a natural isolated strain NRRL1951 derived from an infected cantaloupe because of its high levels of β–lactam production under submerged fermentation conditions. The spontaneous variant NRRL1951 B25 showed almost three times the production levels of *P. notatum* strain NRRL 836 - the first industrial candidate adopted for the tank fermentation method of β–lactam production during the forties of last century. To achieve production of increasing levels of β–lactams and to ease the purification of the product, a comprehensive mutagenesis was applied to the NRRL1951 B25 strain [1]. This included harsh mutagenic techniques like UV irradiation, X-ray, and nitrogen mustard (methyl-bis(β-chloroethyl) amine) treatment as well as selection of the desired spontaneous variants for production and fermentation characteristics. The confidentiality of the industrial CSI programmes prohibits a comprehensive view on the exact mutagenesis steps during the generation of improved penicillin producing strains. Only few intermediate strains can be mapped on the CSI chart with the addressed method of treatment (Fig. 1). However, a major breakthrough in the CSI of NRRL1951 was the selection of the so-called Wisconsin series of improved strains that were derived from the X-1612 (implying X-ray treatment) mutant that was further evolved into the current international laboratory standard strain Wisconsin 54-1255 whose genome sequence became available in 2008 [2]. Currently, Wisconsin 54-1255 is the reference strain for genome, proteome and transcriptome comparative studies [3–7]. The genetic studies and genome sequencing resulted in an understanding of the mechanisms underlying the enhanced β-lactam production, which included the amplification of the penicillin biosynthesis gene cluster [8], an enhanced amino acid production and various aspects of cellular development [2].

Unlike β-lactam biosynthesis, little is known about the consequences of CSI on the secondary metabolism in general. The genome sequence of Wisconsin 54-1255 revealed that in addition to β-lactams, the genome encodes a range of other secondary metabolite gene clusters that have been characterized only to a limited extent. More recently, advances in metabolome techniques systematically revealed a diversity of the secondary metabolites that can be produced by industrial variants of *P. chrysogenum*. For more than 30 of these compounds the genes involved in biosynthesis have been identified and partially characterized. This includes the non-ribosomal peptide synthetases involved in the production of toxins such as roquefortine and meleagrine [9–11], the pigment chrysogine (Ali, unpublished data), siderophores (Samol, unpublished data) and cyclic hydrophobic tetrapeptides [12]. However, this analysis also revealed that during CSI the diversity of the secondary metabolism by improved β-lactam producers has been significantly narrowed. For instance, elimination of the production of yellow pigments like sorbicillin and penitrinic acid, that were contaminants during antibiotic purification, was already achieved at the early stage of CSI [13]. However, the genetic origin of the production of these pigments and their bioactive properties has remained unstudied for long. In recent years many sorbicillin related bioactive compounds were re-identified in *P. notatum* [14], *Trichoderma* [15], and other fungal species isolated from different environments [16]. The broad spectra of activities of sorbicillins range from radical scavenging to cytotoxicity against L5178y leukemic cells, while the actual function and genetic basis for the production of these compounds in *P. chrysogenum* and other filamentous fungi are mostly unknown.

Here we performed a systematic genomic, expression and extracellular metabolome analysis to investigate the impact of CSI on secondary metabolism of *P. chrysogenum*. We demonstrate that CSI resulted in the silencing of many secondary metabolite pathways, whereas others remained unaffected. In addition, we identified the polyketide synthases responsible for the production of a vast group of pigments termed sorbicillinoids that were mutated and transcriptionally silenced at the early stages of the CSI programme.

## Results

### Mutational spread in the *P. chrysogenum* genome during classical strain improvement

To examine the mutational impact of the classical strain improvement (CSI) program on the genome of *P. chrysogenum*, we sequenced the genomes of four *P. chrysogenum* strains. These are the early β-lactam producing strain *P. chrysogenum* NRRL1951, which acts as a reference and two strains in the lineage that signify two different periods of mutagenesis. Here, we define period 1 as the early stage of CSI, using the Wisconsin 54-1255 strain as the hall mark. During this period CSI has had a major impact on the fermentation characteristics and pigment formation. This strain is the current international reference as its genome was sequenced. The second period entails strain DS17690 that has undergone major mutagenesis which amongst others has led to the amplification of the penicillin biosynthetic gene cluster. It is currently studied as a type strain. In addition, the penicillin biosynthesis cluster free strain DS68530 was sequenced that was derived from the latter strain. The absence of the multiple penicillin clusters facilitates the analysis of β-lactam unrelated secondary metabolites present in the extracellular metabolome (Fig. 1). The genomes of the four strains were sequenced using the Illumina HiSeq 2500 platform with near to complete coverage (99.70–99.91 %) (Table 1). Based on a comparison of the respective genomes, 455 SNPs were detected between the NRRL1951 and Wisconsin 54-1255 strain that accumulated during the first period of CSI. Within this set, 215 mutations occurred within coding regions, while 151 non-synonymus, 55 synonymus, 2 termination mutations, 6 frame shifts and one nonsense mutation were identified. During the second period of the CSI the multicopy strain DS17690 additionally obtained 2056 SNPs. This included 1187 non-coding, and 869 mutations within coding regions including 271 synonymus and 558 non-synonymus mutations, three SNPs at the terminal region, 13 frame shifts and 24 nonsense mutations (Table 2, Additional file 1: Table S1).

**Table 1:** Quality statistics of the genome sequencing of *P. chrysogenum* strains within a CSI lineage

Strain	Genome template size (bp)	Number of reads	Sample Yield (in MB)	Average Quality scores (Phred)	Insert size (bp)	Median coverage	Zero template coverage (bp)	Template coverage (%)	Mapped reads (%)
NRRL1951	32228535	38,686,435	11,682	35.20	285	350	28258	99,91	95,44
Wisconsin 54-1255	32228535	34,422,618	10,394	35.20	288	313	27931	99,91	95,78
DS17690	32228535	34,152,533	10,314	35.27	292	306	87244	99,73	95,61
DS68530	32228535	49,691,784	15,006	35.19	302	340	95683	99,70	95,35

**Table 2:** Mutational impact of CSI on a *P. chrysogenum* strain lineage

Strain			Non-coding^a^		Coding^a^			
	Reference	Total		Synonymus	Non-synonymus	Termination mutation	Frameshift	Nonsense
Wisconsin 54-1244	[2]	455	240	55	151	2	6	1
DS17690	[28]	2056	1187	271	558	3	13	24
DS68530	DSM	18	3	7	8	0	0	0

^a^Mutations mapped relative to strain NRRL1951

The use of the advanced next generation sequencing technique to the reference strain Wisconsin 54-1255 resulted in the identification of mismatches with the previously published sequence of this strain [2]. We identified 321 SNPs including 244 non-coding, 10 synonymus and 67 non-synonymus polymorphisms. This included 40 frame shifts, one termination and one nonsense mutation. These newly identified features are the result of the improved quality of the sequencing and correspondingly appeared in the genome assemblies of NRRL1951, DS17690 and DS68530 strain. To simplify the following comparative analysis these SNPs were subtracted from the mutational profiles of the subjected strains. An analysis using GASVPro [17] did not reveal any structural variation between the strains.

In order to assess the distribution of SNPs across genes involved in different cellular processes, we performed a functional classification of the entire gene set of the *P. chrysogenum* Wisconsin 54-1255 reference genome. In order to classify a maximal proportion of genes, we overlapped functional classifications from the COG, KOG and KEGG BRITE databases [18–20] into a single consensus prediction. The complete sets of identified SNPs between NRRL 1951 and Wisconsin 5412-55 and between Wisconsin 54-1255 and DS17690 were mapped to these functional categories. Subsequently, we performed a statistical comparison of the functional distribution of genes affected by the SNPs and the overall functional distribution of all genes in the genome, based on the hypergeometric distribution. Surprisingly, after Bonferroni multiple-testing correction, there was no statistically significant overrepresentation or underrepresentation of mutations in secondary metabolism, amino acid metabolism, transcriptional regulation, or any other functional category (Additional file 2: Table S2). Hence, strain selection seems to have had only very limited control over the mutations accumulated during the CSI, and it is likely that only a minority of the changes introduced by the mutagenesis procedure are responsible for the phenotypic improvements of the strains. The next key challenge was therefore to identify this important minority.

### Distribution of mutations in secondary metabolism gene clusters

**Fig. 2:**
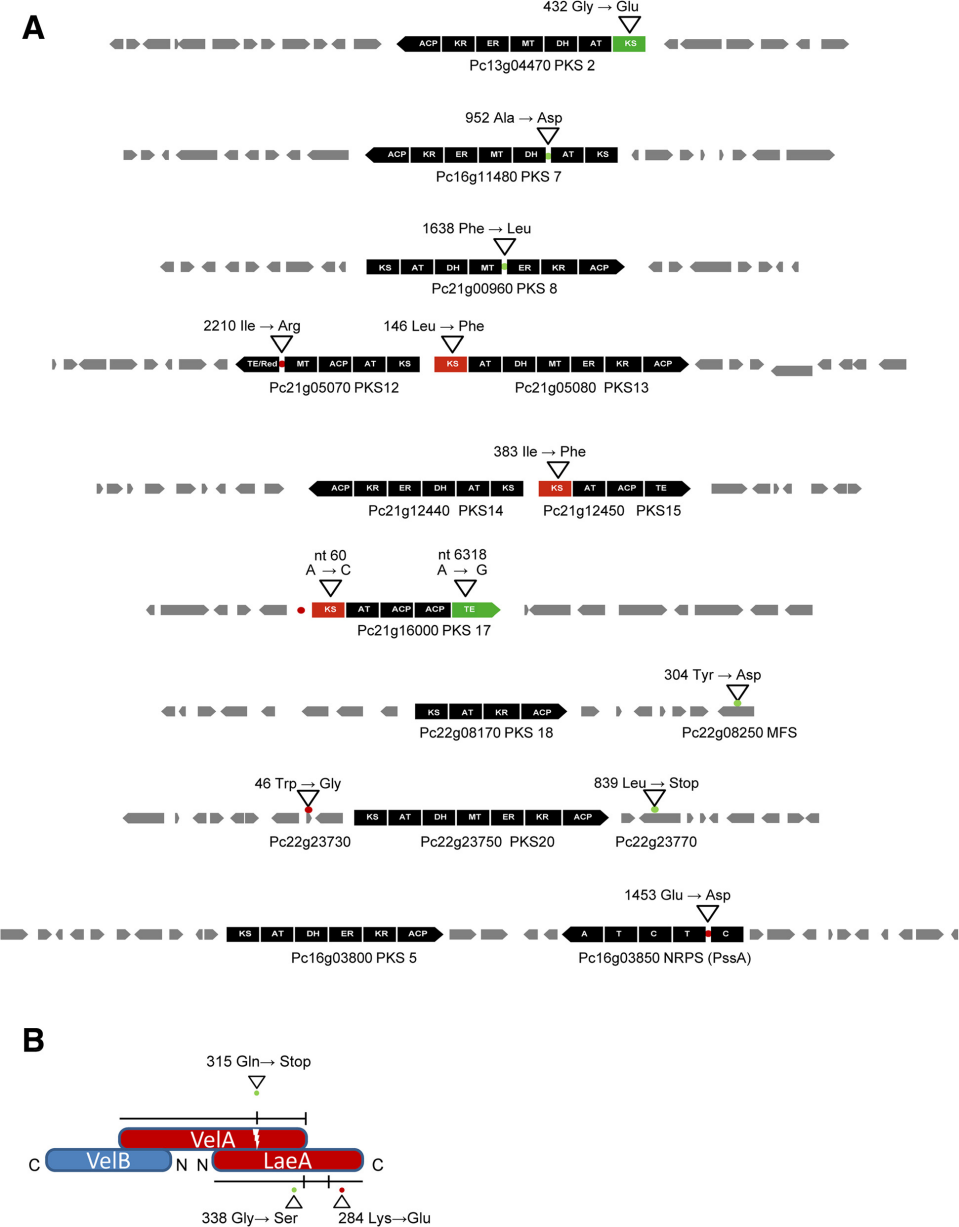
Mutational impact of the CSI program on secondary metabolite gene clusters of *P. chrysogenum.* **a** Mutations acquired by Wisconsin 54-1255 in period 1 of the CSI programme are colored in red. The mutagenic impact in period 2 is shown in green. Mutated PKS and NRPS domains are indicated. The grey arrows represent other genes of the putative gene clusters. Abbreviations used for PKS and NRPS domains: KS, keto-synthase; AT, acyltransferase; DH, dehydratase; MT, methyltransferase; ER, enoyl reductase; KR, ketoreductase; ACP, acyl carrier protein; TE, thioesterase; TE/Red, thioester reductase; A, adenylation; T, thiolation; C, condensation domain. **b** Schematic representation of the Velvet complex proteins VelA, VelB and LaeA with the mutations obtained during the CSI

An inventory was made of mutations that were accumulated in key secondary metabolism genes during the CSI using strain NRRL1951 as a reference. The genome contains 11 NRPS and 20 PKS genes likely involved in secondary metabolite formation. In total, 9 putative secondary metabolite gene clusters accumulated mutational changes during both periods of the CSI (Fig. 2a). This affected 1 NRPS gene and 7 PKS genes. The mutations were all verified by DNA sequencing of the respective gene clusters.

The partially reducing (PR) PKS2 (Pc13g04470) gene cluster is part of predicted cluster of nineteen ORFs [21]. PKS2 shows 87 % identity to an unknown PKS of *P. digitatum* and strong similarity (46 % identity) to the lovastatin diketide synthase LovF of *A. terreus*. Two SNPs occurred at the second period of CSI and resulted in a glycine to glutamic acid substitution at position 432 within the ketosynthase domain of PKS2 that likely has inactivated the enzyme. The gene cluster of PR PKS7 (Pc16g11480) is predicted to consist of 18 ORFs [21]. A homologous cluster of five genes is found in the genome of *A. fumigatus*, but has no assigned function. PKS7 carries the SNP resulting in an alanine to aspartic acid substitution at position 952 located at the acyl transferase/dehydratase inter-domain region. This mutation was acquired in the second CSI period and presumably does not affect the function of the neighboring domains. The highly reducing (HR) PKS8 (Pc21g00960) is homologous (54 % identity) to an uncharacterized PKS enzyme of *A. flavus* and belongs to a putative cluster of 17 genes [21]. A single mutation resulted in a phenylalanine to leucine substitution at position 1638 in the C-(Met)/dehydratase inter-domain region and occurred in the second period of CSI. A further gene cluster contains two PKSs encoding genes HR PKS14 (Pc21g12440) and non-reducing (NR) PKS15 (Pc21g12450) and consists of 18 ORFs [21]. A mutation within the ketosynthase domain of PKS15 occurred during the first CSI period and resulted in isoleucine to phenylalanine substitution at position 383. A NR PKS encoded by Pc21g16000 (PKS17) shows similarity to the *A. nidulans* wA and *A. fumigatus* PksP naphthopyrone synthases. These enzymes are involved in the biosynthesis of a precursor for the formation of a conidial pigment [2]. A similar gene cluster of 11 ORFs is present in the genomes of *A. niger* and *A. kawachii*. Two nucleotide substitutions were detected within PKS17 causing no effect on the amino acid sequence. A third mutation is located within the terminator region of the PKS gene and is represented by an A to C nucleotide substitution. The actual impact of these mutations on pigment formation is unclear as the green coloring of the conidia was not affected during the CSI.

The gene cluster of PR PKS18 (Pc22g08170) shows 82 % identity to the 6-methylsalicylic acid synthase from *P. griseofulvum* and contains 16 ORFs. The *P. griseofulvum* homolog is involved in patulin biosynthesis and requires additional enzymatic conversions like decarboxylation, two hydroxylation and oxidation reactions [22]. In *P. chrysogenum* these enzymatic steps are presumably provided by the aminohydrolase and two cytochrome P450 enzymes encoded within the cluster. However, the essential gene encoding the isoepoxidon dehydrogenase cannot be identified in the genome of *P. chrysogenum* supporting the notion that this fungus does not produce patulin [2]. A single mutation was obtained during the second period of CSI and this affected a putative MFS transporter (Pc22g08250) encoding gene introducing a tyrosine to asparagine substitution at position 304. Finally, two neighboring, oppositely oriented NR PKS12 (Pc21g05070) and HR PKS13 (Pc21g05080) that belong to a gene cluster that potentially is related to sorbicillinoid biosynthesis, received SNPs in both coding sequences. PKS12 acquired an isoleucine to arginine substitution at C-(Met)/TE inter-domain region, while PKS13 obtained a mutation in the KS domain where leucine in position 146 is substituted by a phenylalanine. Both mutations were obtained at the first period of the CSI and thus were inherited already by strain Wisconsin 54-1255. The associated gene cluster is predicted to contain 18 ORFs [21].

The only NRPS gene that accumulated SNPs concerns PssA (Pc16g03850) that is involved in siderophore coprogen B biosynthesis under the iron depleted growth conditions. At the first CSI period the PssA encoding gene was mutated causing a glutamic acid for aspartic acid substitution at position 1453 within a non-conserved region. PssA is involved in the production of coprogen that still can be produced by the improved β-lactam producing strains (Samol, unpublished data). Therefore, this mutation apparently did not inactivate the enzyme.

### Mutational changes within the Velvet complex

The Velvet complex is involved in the regulation of sexual development and secondary metabolism as demonstrated in various filamentous fungi including *P. chrysogenum* [3, 6, 7]. Since it is a multisubunit complex, we examined the impact of CSI on the core component of the Velvet complex. The *P. chrysogenum* orthologs LreA (Pc20g08380), LreB (Pc22g02540), FphA (Pc06g00040), KapA (Pc21g01970), VelB (Pc22g22320), and VosA (Pc22g06890) were all identical in the genomes of strains NRRL1951, Wisconsin 54-1255 and DS17690. However, mutational changes were found in Pc16g14010 and Pc13g13200 genes that encode LeaA and VelA, respectively. In the first period, the *leaA* gene acquired a nucleotide substitution at position 850 (T to C) which resulted in a substitution of lysine for glutamic acid at position of 284 of the protein. A second mutation occurred in period 2 and caused a nucleotide change at position 1012 (C to T) causing a glycine to serine substitution at position 338. In addition, a mutation was identified in the VelA encoding gene that occurred at the second CSI period. A C to T substitution at nucleotide position 943 caused a stop codon resulted in the formation of a truncation of the VelA protein from 562 to 315 amino acids long. These data indicate that the velvet complex has been a major target in the CSI, and this likely has impacted the expression of secondary metabolite encoding genes (Fig. 2b).

### Expression of the secondary metabolism gene clusters

**Fig. 3:**
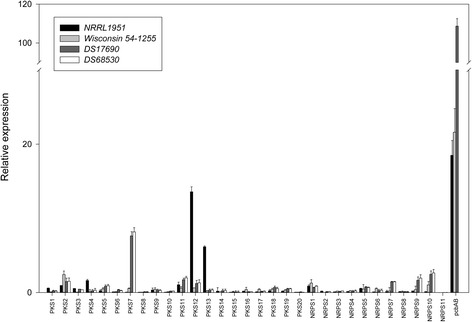
Comparison of secondary metabolite gene expression in strains NRRL1951, Wisconsin 54-1255, DS17690 and DS68530. Expression data was obtained with RNA isolated after 5 days of fungal growth. The error bars indicate the standard error of the mean of two biological samples including technical duplicates

**Fig. 4:**
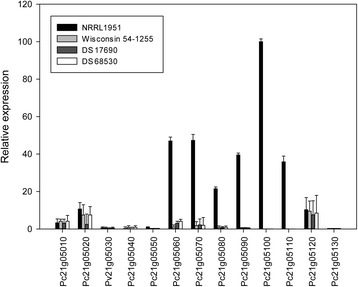
Relative expression of the putative gene cluster of sorbicillinoid biosynthesis in strains NRRL1951, Wisconsin 54-1255, DS17690 and DS68530. The transcriptional silencing of seven genes (Pc21g05050 – Pc21g05110) belonging to the putative sorbicillinoid gene cluster is shown. Expression data was obtained with RNA isolated after 5 days of fungal growth. The error bars indicate the standard error of the mean of two biological samples including technical duplicates

Since the velvet complex and many secondary metabolism genes acquired mutations during the CSI, the expression of the latter genes may have vastly changed along the lineage of strains. Therefore, we examined the expression of all 20 PKSs and 11 NRPS encoding genes in strains NRRL1951, Wisconsin 54-1255, DS17690 and the penicillin cluster free strain DS68530 (Fig. 3). Herein, the fungi were grown for 5 days and total RNA was isolated for qPCR analysis of the respective genes. The expression of γ-actin gene (Pc20g11630) was used as reference for normalization. The observed Ct values for the amplified γ-actin gene from the cDNA of NRRL1951, Wisconsin 54-1255, DS17690 and DS68530 were 19.9 ± 0.12; 20.11 ± 0.18; 20.48 ± 0.25 and 20.30 ± 0.18 respectively. This demonstrates nearly identical expression of the reference gene in the analyzed strains. The obtained expression profiles clearly indicates that the expression of the ACV synthetase, the key enzyme of β-lactam biosynthesis was dramatically enhanced upon CSI of Wisconsin 54-1255 as expected from the amplification of the β-lactam biosynthesis genes in strain DS17690 that contains 8 copies of this penicillin cluster. With the other NRPS and PKS genes, there were no marked expression changes except for three PKS genes. The two highly expressed PKS genes Pc21g05070 (PKS12) and Pc21g05080 (PKS13) were dramatically down regulated in Wisconsin 54-1255 and the derivatives DS17690 and DS68530. The NR PKS12 exhibits 43 % identity to citrinin polyketide synthase of *Monascus purpureus* while HR PKS13 is homologous to lovastatin diketide synthase LovF of *A. terreus* [2]. In addition, the cluster contains genes encoding a putative regulator (Pc21g05050), a monooxygenase (Pc21g05060), a transcriptional factor (Pc21g05090), a MFS transporter (Pc21g05100) and an oxidoreductase (Pc21g05110). All five genes of this putative cluster are also present in the genome of *Trichoderma reesei* and arranged in one locus. An identical gene cluster in the marine isolate of *P. chrysogenum* E01-10/3 has been previously associated with the biosynthesis of sorbicillactones A and B and thus by analogy, the aforementioned gene cluster may encode the biosynthesis pathway of yellow pigments sorbicillinoids in *P. chrysogenum* [23]. There is a significant decrease in expression of the seven genes of this cluster in the improved strains, whereas expression of flanking genes that are likely not part of the cluster was unaltered (Fig. 4). In addition, expression of PKS7 was increased substantially upon CSI of the NRLL1951 strain. Despite the significant overexpression of this PKS in the improved penicillin producing strain, the polyketide product has not yet been identified.

### Comparative metabolomic profiling of *P. chrysogenum* strains

**Fig. 5:**
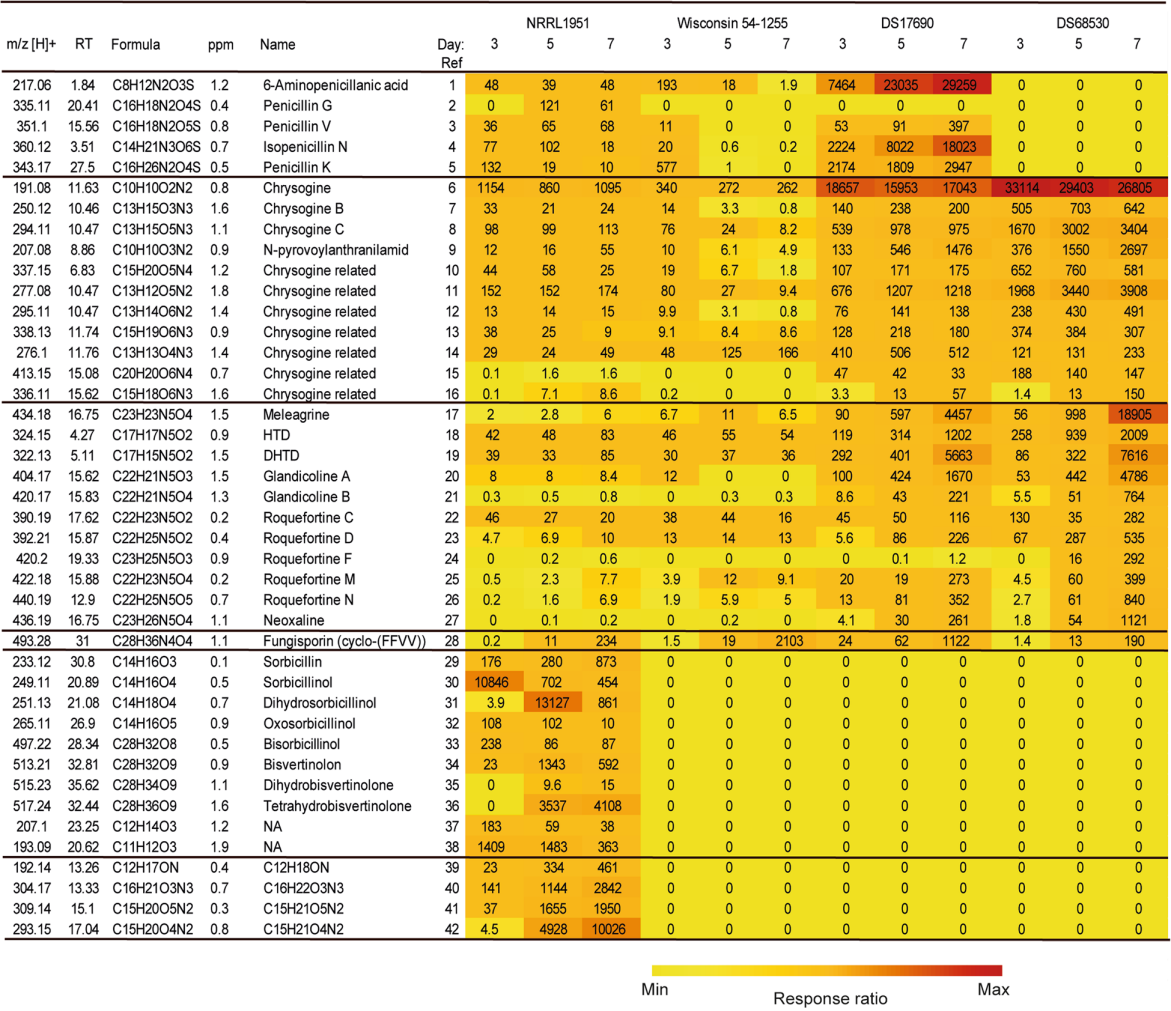
Response ratio of secondary metabolite concentrations present in the culture broth of strains NRRL1951, Wisconsin 54-1255, DS17690 and DS68530. Cell coloring corresponds to the internal standard corrected peak area of the detected metabolites in the culture broth after 3, 5 and 7 days of growth. The mass to charge ratio (m/z) of the protonated metabolites, retention time (RT) and empirical formulas are indicated. Abbreviations: HTD, histidyltryptophanyldiketopiperazine; DHTD, dehydrohistidyltryptophanyldi-ketopiperazine; NA, metabolites without structural data

Next, we examined the extracellular metabolome focusing on secondary metabolites excreted into the spent culture medium. LC-MS and LC-MS/MS were used for identification. In this analysis, five β-lactams were detected in the growth media of all strains, except for the penicillin biosynthesis cluster-free strain DS68530 (Fig. 5). 6-APA (compound 1), penicillin G (2), V (3), isopenicillin N (4) and penicillin K (5) were identified by their expected molecular masses and on the basis of their calculated empirical formulas and by the use of standards, except for penicillin K. 6-APA and isopenicillin N were produced in the early stages of fermentation. As expected, the levels of these compounds were relatively low for the NRRL1951 strain, increased in strain Wisconsin 54-1255, and dramatically increased in DS17690, in particular during the late growth phase. 6-APA, isopenicillin N and the naturally produced penicillin K were the main products. Since the growth conditions did not involve any feed with the commercially used side chain precursors, penicillin G and V were presented very low amounts compared to other penicillins.

Another group of compounds are the chrysogine related metabolites. This yellow pigment is produced in a biosynthetic pathway that has not yet been completely characterized, but at least four structurally resolved compounds can be identified including chrysogine (6), N-acetylalanylanthranilamide (chrysogine B) (7), N-malonylalanylanthranilamide (chrysogine C) (8) and N-pyrovoylanthranilamide (9), as well as 7 related but structurally unresolved molecules (10-16). These metabolites are already found in the culture broth after 3 days. Chrysogine was produced at high levels by the DS17690 strain, but in particular in the related penicillin cluster free DS68530 strain, production was at the highest levels.

A further class of compounds are the roquefortine and meleagrin related metabolites that are toxins [9–11]. The precursors for these toxins are (histidyltryptophanyldi-ketopiperazine (HTD) (18) and dehydrohistidyltryptophanyldi-ketopiperazine (DHTD) (19) that are only produced at low levels by strain NRRL 1951 and Wisconsin, but at substantial higher levels during the late growth phases by the DS17690 strain. Production further increased in the DS68530 strain. The same observation was made for the precursors of this pathway like glandicoline A-B (c20-21), roquefortine C, D, F, M, N (22-26) and neoxalin (27), as well as the final product meleagrin (17). It thus appears that like with the chrysogine, period 2 of the CSI has resulted in an enhanced production of roquefortines.

**Fig. 6:**
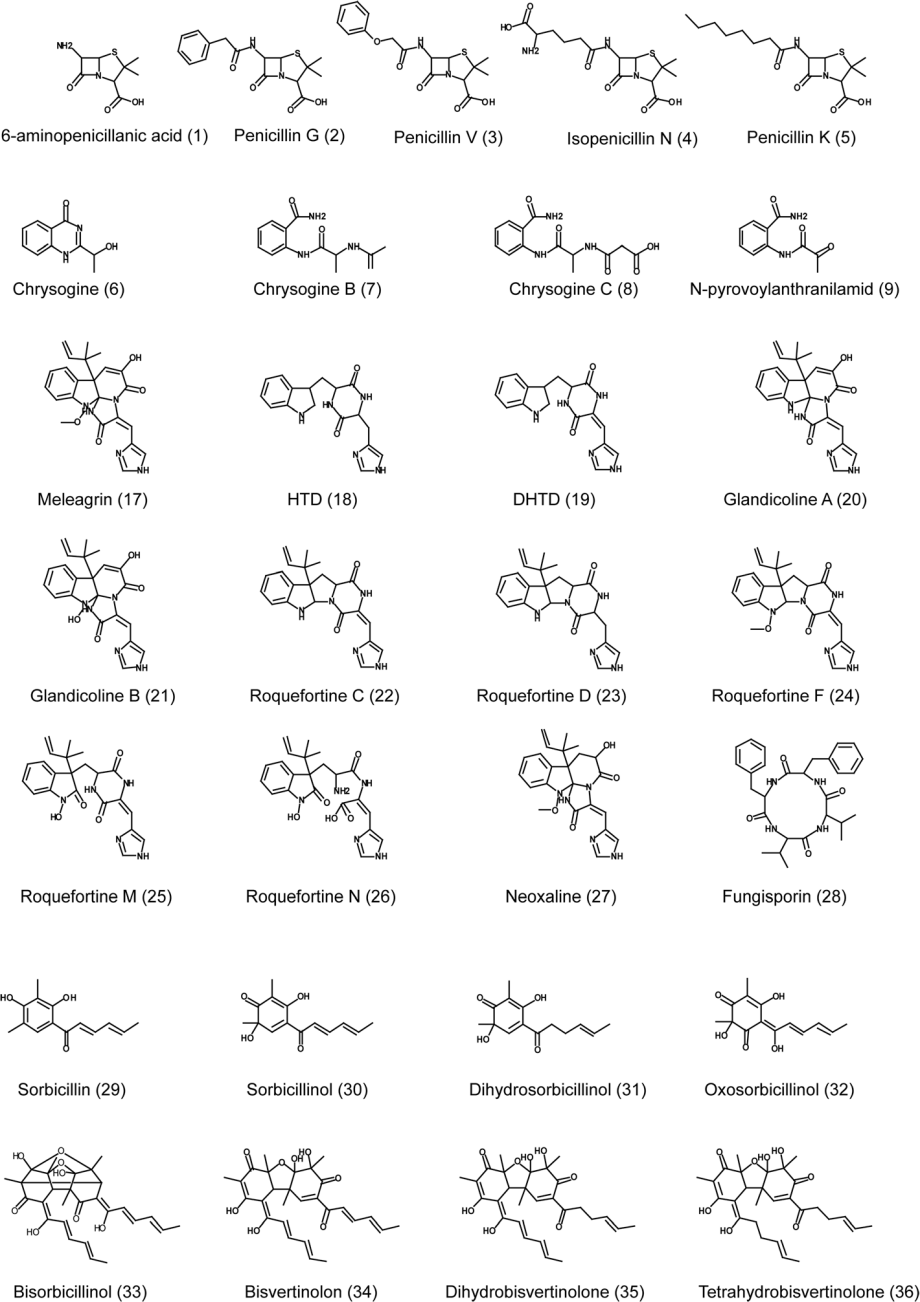
Metabolites characterized in this study. β-lactam related compounds (1-5), the intermediates of chrysogine pathway (6-9), roquefortine/meleagrin related compounds (17-27), the representative of the cyclic tetrapeptides - fungisporin (28) and the sorbicillinoids (29-36)

In the analysis, a unique group of 16 metabolites were identified in the culture broth of NRRL1951 that were absent in the other more evolved strains. Based on their exact masses and their MS spectra, empirical formulas could be assigned to these molecules with less than 2 ppm accuracy. Two groups of compounds could be discriminated (Fig. 5). The first group includes eleven molecules related to sorbicillins that are known to be produced by natural isolates of *P. chrysogenum*. Both, monomeric and dimeric sorbicillinoids were identified in the culture broth during seven days of culturing. The identified compounds fit to the proposed biosynthesis pathway [23, 24]. Major molecules are sorbicillin (29) and the derivative sorbicillinol (30) that already occur after 3 days of fermentation. Although dihydrosorbicillin was not detected, the accumulation of its oxidized derivative dihydrosorbicillinol (31) occurred after five days of cultivation. Oxosorbicillinol (32), and the oxidized compound bisvertinolon (34) (dimerization reaction with sorbicillinol) were identified. Further dimers were bisorbicillinol (33), dyhydrobisvertinulone (35) and tetrahydrobisvertinolone (36). In addition, two uncharacterized metabolites with a short (C11 and C12) carbon chain (37-38) were observed. The second group included four, presumably nitrogen containing compounds (39-42) (Fig. 6).

## Discussion

Here we have examined the effect of the classical strain improvement (CSI) program on secondary metabolism by *P. chrysogenum*. Combined metabolome, transcriptome and genome profiling approaches were applied to a lineage of β-lactam producing strains. This concerned the natural isolate strain NRRL1951, the current laboratory reference Wisconsin 54-1255 and the high penicillin producer DS17690 which carries multiple copies of the penicillin biosynthesis cluster. In addition, a DS17690 derivative, which lacks the penicillin clusters, i.e., strain DS68530 was included in this analysis (Fig. 1). The genomes of the subjected strains were sequenced using the Illumina next generation technique that allowed us to determine the mutational impact on *P. chrysogenum* during CSI.

Of the 20 PKSs and 11 NRPS present in *P. chrysogenum,* the majority remains uncharacterized and has not been associated to the production of specific secondary metabolites. It is generally believed that many of these genes and gene clusters are transcriptionally silent or inactive. The introduction of the parental isolate NRRL1951 in our analysis revealed a number of mutations inherited by the current international laboratory standard strain Wisconsin 54-1244 during the first period of CSI. Further comparative analysis showed the accumulation of additional mutations during the evolvement of the industrial penicillin cluster multi-copy strain DS17690. Out of the 11 NRPS and 20 PKS associated gene clusters present in the genome, one NRPS and 7 PKS gene clusters accumulated mutational changes during both periods of the CSI. Three amino acid substitutions were located within the essential ketosynthase domains of PKS2, PKS13 and PKS15 that presumably led to a functional inactivation (Fig. 2a).

Significant changes of the secondary metabolite genes expression between the CSI derived mutants as well as the mutational impact on transcriptional factor encoding genes indicates that the regulation of secondary metabolism has been intensively altered during the CSI (Fig. 3). The three proteins VelA, VelB and LaeA of the velvet complex represent an important regulatory element in filamentous fungi. In *A. nidulans,* this heterotrimer has been associated with both cell development and secondary metabolism providing the first evidence on the regulatory cross-talk between these phenomena in filamentous fungi. The current structural model of the Velvet complex is derived from protein interaction studies performed in *A. nidulans* [25]. VelA is a scaffolding protein essential for recruiting VelB and LaeA. VelB interacts with the N-terminus of VelA while the C-terminus interacts with LaeA. It is important to emphasize that all recently published studies on the characterization of the Velvet complex proteins in *P. chrysogenum* were performed in strains derived from the CSI, i.e., the eight copy penicillin gene cluster strain DS17690 derived from Wisconsin 54-1255 and the two copy penicillin gene cluster strain P2niaD18 obtained during the alternative Panlabs strain improvement program [8]. Both overproducers originate from the parental NRRL1951 isolate but the P2 series diverged early at the CSI prior the generation of Wisconsin 54-1255. A pronounced phenotypic effect of the deletion of VelA and LaeA on β-lactam titers (up to 80 % decrease) in conjunction with a dramatic transcriptional deregulation of up to 10 % of genes present in the genome was demonstrated for the Panlabs derived P2niaD18 strain [3, 26]. In contrast, the penicillin gene expression and secondary metabolite production in DS17690 strain exhibited only moderate responses to the deletion of VelA and LaeA. The latter study, however, was performed under glucose-limited chemostat growth conditions, and it was suggested that the growth conditions weakened the phenotype [7]. However, since the Velvet complex has been repetitively targeted during the improvement of the DS17690 strain (Fig. 2b) this likely caused the weak phenotypes of the respective deletion strains. Indeed, the P2niaD18 strain [27] shows no alterations of the Velvet complex encoding genes and must therefore have remained functionally intact. Specifically, in LeaA, we identified two mutations resulting in amino acids changes while in VelA, in DS17690 strain a stop codon is introduced resulting in a C-terminal truncation by about 247 amino acids. VelA plays a primary role in the complex assembly where the C-terminus that was lost, is required for interaction with LaeA. Therefore, the velvet complex of the improved DS17690 is likely functionally impaired and this must have contributed to an improvement of the β-lactam production presumably as a collateral effect, increased or decreased expression of other secondary metabolite genes. In addition, pathway specific transcriptional regulation of secondary metabolism is crucial. We identified 19 mutated proteins specifying Zn2Cys6 binuclear cluster DNA-binding domains typical found in transcription regulators like GAL4 and six genes encoding proteins that are homologous to fungal specific transcription factors. Additionally, 29 transporter related genes were mutated during the CSI, eight ABC transporters and 15 transporters belonging to the major facilitator superfamily (MFS) (Supplementary XCL1). One of the identified MFS Pc22g08250 belongs to the putative secondary metabolite gene cluster harboring a PKS gene (Pc22g08170) that can be linked to the biosynthesis of patulin-like metabolites in *P.* chrysogenum [2]*.*

The mutational and expression effects of CSI led to the significantly alterations in the secondary metabolite profile among the analyzed *P. chrysogenum* strains. Previously, genes and gene clusters have been identified for the production of roquefortine/meleagrine [11], cyclic tetrapeptides [12] and chrysogine (Ali, unpublished data) that all arise from the activity of NRPSs. By high-resolution mass spectrometry we generated a specific metabolite profile for each of the strains and demonstrate significant alterations in the pattern of the production of these secondary metabolites (Fig. 5). Importantly, during the CSI and with the onset of strain DS17690, the ability to produce toxins such as roquefortine/meleagrine and chrysogen increased. However, the transcriptional levels of the *roqA* and *chyA* genes was not significantly changed in the lineage of strains. Considering that the biosynthesis of these secondary metabolites is strongly dependent on nitrogen metabolism, it seems likely that the improvement in amino acid production [2] in the higher β-lactam producers is the indirect cause of this phenomenon. Moreover, the highest levels of these secondary metabolites where found in the DS68530 strain in which the multiple penicillin gene clusters have been removed [28]. This strain is genomically identical to the parental strain DS17690, also showing the same expression levels and timing of the expression of the corresponding NRPS encoding genes. Therefore, the observed increase in the aforementioned nonribosomal peptide products strongly supports our notion that their enhanced production is because of a re-diversion of nitrogen metabolism.

During the CSI, also the production of a suite of secondary metabolites was lost. Early *P. chrysogenum* isolates produced yellow pigments that were eliminated early on during CSI as they were found to be colored contaminants during antibiotic production. However, the genetic origin of pigment formation has remained obscure. Our secondary metabolite profiling indicates a group of structurally related compounds that are specifically produced by the NRRL1951 strain but are absent in the fermentation broth of Wisconsin 54-1255, DS17690 and DS68530. The empirical formulas generated based on mass accuracy (ppm < 2) correspond to known hexaketide yellow pigments termed sorbicillinoids. The production of sorbicillin, sorbicillinol, dihydrosorbicillinol, oxosorbicillinol, bisvertinolone, bisorbicillinol, and dihydrobisvertinolone was detected. We found that accumulation of the pathway precursors sorbicillin and sorbicillinol by a three days grown culture correlates with high levels of expression of a single PKS gene cluster in the NRRL1951 strain. qPCR analysis revealed the transcriptional silencing of the entire gene cluster of seven genes although expression of the flanking genes remained unchanged. Additionally, the genome sequencing analysis revealed two mutations accumulated in the two PKS encoding genes of the aforementioned cluster at the beginning of the CSI program. NR-PKS12 acquired an isoleucine to arginine substitution at the C-(Met)/DH inter-domain region. The neighboring oppositely oriented HR-PKS13 encoding gene obtained a mutation causing a leucine to a phenylalanine substitution in position 146 within the KS domain of the enzyme. The proposed model for sorbicillin biosynthesis, as the hexaketide precursor for all sorbicillinoid derivatives, foresees the function of a pair of NR-PKS and HR-PKS enzymes. The triacetate precursor is formed by HR-PKS following the non-reductive carbon chain extension by the second NR-PKS to form hexaketide. Taken together our results suggest this PKS gene cluster is responsible for the biosynthesis of sorbicillinoids in the early producer NRRL1951. The mutations found within the PKS gene cluster combined with the transcriptional silencing of the entire gene cluster shown for derivative strains Wisconsin 54-1255 and DS17690, demonstrates a multi-step complex eliminating effect of the CSI on the biosynthesis of these yellow pigments in *P. chrysogenum*. Importantly, the functional characterization of the aforementioned gene cluster through direct targeting of the PKS genes has not yet been achieved [23, 29], and will be subject of future work.

## Conclusions

Our study concerned a combined genome, transcriptome and metabolome analysis of improved β-lactam producing strains of *P. chrysogenum* with a focus on secondary metabolism. Whereas several secondary metabolite genes were targeted by mutagenesis, overall there was not a single specific functional group of genes that was specifically mutagenized. Mutations appear to be randomly spread across the chromosomes and this is likely the result of the rather undirected approach used during CSI. The results suggest that the improved titers of the β-lactams achieved during the CSI are only partially reflected by the broad mutagenic impact of the CSI program. Whereas mutagenesis was random, the selection was focused on specific features such as the increased β-lactam titers; morphological aspects as well the fermentation characteristics. Thus, the strains collected many mutations that do not contribute to these features. The elevated production of nonribosomal peptide products such as chrysogine and roquefortine/meleagrin likely emerged from the enhanced nitrogen metabolic flux in improved penicillin producing strains. A group of yellow pigments including sorbicillinoids has been eliminated at the early stages of CSI. Production of sorbicillinoids is likely associated with a PKS gene cluster of seven genes that was transcriptionally silenced during the CSI and that acquired mutations at functionally essential PKS domains. These finding indicate a complex mechanism underlying the elimination of the production of sorbicillinoids from the secondary metabolism of *P. chrysogenum* and provide insight in the molecular basis of biosynthesis of this interesting group of bioactive compounds.

## Methods

### Strains and culture conditions

The parental *P. chrysogenum* isolate NRRL1951, Wisconsin 54-1255, the penicillin overproducer DS17690 (multi-copy penicillin gene cluster) and the penicillin free strain DS68530 (zero-copy penicillin gene cluster) used in this study were kindly provided by DSM Biotechnology Center. For secondary metabolite analysis and fungal RNA extraction, conidia were pre-grown in liquid YGG medium [30] for 24 h before transfer into minimal metabolite medium (SMP) (glucose, 0,5 g/l; lactose, 75 g/l; urea, 4.0 g/l; Na_2_SO_4_, 4.0 g/l; CH_3_COONH_4_, 5.0 g/l; K_2_HPO_4_, 2.12 g/l; KH_2_PO_4_, 5.1 g/l). All cultivations were performed at 25 °C in semi dark conditions using 100 ml shaking flasks with a volume 25 ml of SMP medium.

### Genome analysis

Total genomic DNA (gDNA) was isolated from the mycelium after 4 days of cultivation in YGG liquid medium followed by enzymatic protoplasting with *T. harzianum* lytic enzyme using the adapted yeast genomic DNA isolation protocol (Promega). The next generation sequencing of the strain NRRL1951, Wisconsin 54-1255, DS17690 and DS68530 was performed using the Illumina HiSeq 2500 platform (BaseClear, Leiden, The Netherlands). FASTQ compressed files of the paired-end sequence reads were generated for each of the subjected strains using Illumina Casava pipeline version 1.8.3. Quality assessment was based on data passing the Illumina Chastity filtering. Subsequently, adapters were removed and the second quality assessment of the remaining reads was performed using the FASTQC quality control tool version 0.10.0. The resulted Average Quality scores (Phred > 35) was determined for each of the analyzed genomes (BaseClear, Leiden, The Netherlands). The alignment of the reads was generated with DNASTAR using the available sequence of Wisconsin 54-1255 as the reference. High quality reads of the genomes were obtained with near to complete coverage (99.70 – 99.91 % and median coverage 306-350) (Table 1). The resulted BAM files were used to obtain a list of single nucleotide polymorphisms (SNPs) with SeqManNGen11(DNASTAR) tool using 90 % of the SNP carrying reads as the cutoff parameter for selection. The structural variation analysis was performed with GASVPro [17].

### LC-MS analysis

Metabolite analysis was performed using the Accella1250™ HPLC system coupled with the benchtop ES-MS Orbitrap Exactive™ (Thermo Fisher Scientific, San Jose, CA). A sample of 5 μL was injected onto a Shim-pack XR-ODS™ C18 column (3.0 x 75 mm, 2.2 μm) (Shimadzu, Japan) operating at 40 °C and flow rate of 300 μL/min. The linear gradient began with 90 % of solvent A (100 % water) and 5 % of solvent C (100 % acetonitrile) starting after 5 min of isocratic flow. The first linear gradient reached 60 % of C at 30 min, and the second 95 % of C at 35 min. A washing step for 10 min at 90 % of solvent C was followed by column equilibration for 15 min at the initial isocratic conditions. Solvent D (2 % formic acid) was continuously used to maintain a final 0.1 % of formic acid in the system. The column fluent was directed to the Exactive™ ES-MS Orbitrap operating at the scan range (m/z 80 – 1600 Da) and switching to positive/negative modes. Voltage parameters for the positive mode was 4.2 kV spray, 87.5 V capillary and 120 V of tube lens. Voltage parameters for the negative mode was 3 kV spray, -50 V capillary, -150 V tube lens. The capillary temperature was 325 °C, sheath gas flow 60 a.u., and auxiliary gas was off to maintain higher detection sensitivity for both positive and negative modes during analysis. The obtained raw files were processed using SIEVE software (Thermo Fisher Scientific, San Jose, CA). The resulting peak tables were used as the target list in which each feature was integrated in every individual sample. For more accurate integration, the discovered features were selected and transferred into Excalibur 2.1 (Thermo Fisher Scientific, San Jose, CA) processing tool. Auto-integration of the peaks was performed using base peak traces in mass range 10 ppm with a window of 60 s.

### LC-MS sample preparation

For secondary metabolite analysis, samples of the culture broth were taken after 3, 5 and 7 days of growth in SMP medium. After centrifugation at 14,000 rpm for 10 min at 4 °C, 100 μL of the supernatant was supplemented with 16 μL of reserpine (600 μmol/mL) as the internal standard. After filtration using 0.2 μm polytetrafluorethylene (PTFE) syringe filters samples were quickly frozen in liquid nitrogen and stored at -80 °C.

### qPCR analysis

Total RNA was isolated after 3 and 5 days of culturing of the fungal strains on SMP medium. The Trizol™ (Invitrogen) extraction method was used, with additional DNAse treatment using the Turbo DNA-free™kit (Ambion). The total RNA concentration was measured with a NanoDrop ND-1000™. For the synthesis of cDNA by iScript™ cDNA synthesis kit, 500 ng of RNA per reaction was used (Bio-Rad). Primers were designed around the introns in order to be able to discriminate between amplification on gDNA and cDNA and are listed in Table 3. For expression analysis, the γ-actin gene was used as a control for normalization. A negative reverse transcriptase (RT) control was used to determine the gDNA contamination in the isolated total RNA. The expression levels were analyzed for two biological samples that were split into technical duplicates. Measurements were done with a MiniOpticon™ system (Bio-Rad) using the Bio-Rad CFX™ manager software, with which the threshold cycle (CT) values were determined automatically by regression. The SensiMix™ SYBR Hi-ROX kit (Bioline) was used as a master mix for qPCR. The following thermocycler conditions were applied: 95 °C for 10 min, followed by 40 cycles of 95 °C for 15 s, 55 °C for 30 s, and 72 °C for 30 s. Subsequently, a melting curve was generated to determine the specificity of the qPCRs.

**Table 3:** Oligonucleotide primers used for qRT-PCR

Primer name	Gene	Sequence 5′-3′
PKS1F	Pc12g05590	GCTACAGCCCTGACGCCATGG
PKS1R	Pc12g05590	CTGCGCAGGTCTACATCGGTACC
PKS2F	Pc13g04470	CCGAAGATGCCGGCGACGG
PKS2R	Pc13g04470	CGCTGGTCTGCGATGTGGCC
PKS3F	Pc13g08690	CGAGAGACCAGGATAAGGTTCTTGGC
PKS3R	Pc13g08690	GGTGGTCTGTCACCACTCTTCCC
PKS4F	Pc16g00370	CATGGTCAGCACCCTCAGTGCC
PKS4R	Pc16g00370	CCAGGTCAGGCGTCGTACGC
PKS5F	Pc16g03800	CGGGTGCTGCATAGATGTACTACGC
PKS5R	Pc16g03800	GCTGGCCACGGAAGACAACGC
PKS6F	Pc16g04890	CCTATTCGCGCCCTGATTATGGGC
PKS6R	Pc16g04890	CGAGATTTGTCTTCACAGAACCCACC
PKS7F	Pc16g11480	CACGATTTTAGCAAGTCAACCAGCGCG
PKS7R	Pc16g11480	CTCGCTCTCCCAGAATGTCAAGGC
PKS8F	Pc21g00960	GCCACACTCATCGGCACCACG
PKS8R	Pc21g00960	GCTCCACAGAGCAACCAACCCG
PKS9F	Pc21g03930	GACGTGGCCGGTGATGCCG
PKS9R	Pc21g03930	GCGATGTTGCGGACGAGGCC
PKS10F	Pc21g03990	CAGCGCCGAGTCCTACAGCC
PKS10R	Pc21g03990	GTGGACCTTGGAGGATGTCTTGC
PKS11F	Pc21g04840	CCTTGACGAATATCCGCACTCCG
PKS11R	Pc21g04840	CAAGCCACAGCTGATGAAGCGC
PKS12F	Pc21g05070	GTCGGAGGCAATTCGGGAAGGC
PKS12R	Pc21g05070	GCAAAGTTCCACCACAATGCCGCG
PKS13F	Pc21g05080	CCGAGGATCTCCGCCAGGC
PKS13R	Pc21g05080	GGTTGTGCAGGTTCCAGGTGCC
PKS14F	Pc21g12440	GCACCACCATCAGCCAAAGCATACC
PKS14R	Pc21g12440	CCGAGGTCCATTGGAACTATGCGC
PKS15F	Pc21g12450	CCAGTTGTCTGCAGCCGGCC
PKS15R	Pc21g12450	GCCCAGATCACCGCCGTACG
PKS16F	Pc21g15160	CAGCCGCGTAGTTTGCCTGGC
PKS16R	Pc21g15160	GCACAGTGTGCTGAGGTTACGGC
PKS17F	Pc21g16000	CTTGTCATCAGCAGCCCAGAGG
PKS17R	Pc21g16000	CAATTTGCGGTGGCTGAGACGC
PKS18F	Pc22g08170	GGTTGATACTCCTGGGACTGAATACAG
PKS18R	Pc22g08170	GCTGCTGTGGATCCATCTGCTCG
PKS19F	Pc22g22850	CGGTCAACCAGGGATCCAACTGC
PKS19R	Pc22g22850	CTGAAGCGGTCTCTGTGTGGCC
PKS20F	Pc22g23750	CGGTAATGTCCAGCTGGCACTCG
PKS20R	Pc22g23750	CTTCAGGCACTTCTGTACCGGG
NRPS1F	Pc13g05250	GCAGACCTGTATCCATCGCAA
NRPS1R	Pc13g05250	GGAGGCAAGTGAAGGTGTGTT
NRPS2F	Pc13g14330	GCGACAGCCGCCGGAGTAACTATGG
NRPS2R	Pc13g14330	GAGAGACGGGGACACGCGTGATG
NRPS3F	Pc14g00080	ACGTACGCTCGAGCTGGACT
NRPS3R	Pc14g00080	GCCGTCGCGTTGATAATTGG
NRPS4F	Pc16g03850	TGGTTGAAAGAGGGCAGTCTC
NRPS4R	Pc16g03850	CGCGAACATACACAACACCAC
NRPS5F	Pc16g04690	CTTTCCAGAACAGTTGGCTGGT
NRPS5R	Pc16g04690	GCTGCATCTTACCCAGGTAATTG
NRPS6F	Pc16g13930	CCACCCTTGTTCAGCCGCTGAATTCC
NRPS6R	Pc16g13930	GGACGAGGCGAACAACATCGGAC
NRPS7F	Pc21g01710	GCTATCTCGGTGGAGGATCTTCTGTCC
NRPS7R	Pc21g01710	GTGCTGCTGAGAACACGGGATTGT
NRPS8F	Pc21g10790	GTGAGGCAGCTTTGTTCAACACCATT
NRPS8R	Pc21g10790	TTCTGCAGCAGGCTGTCGGCCTGAG
NRPS9F	Pc21g12630	GAGCCAACTCTGTTGTCTACG
NRPS9R	Pc21g12630	CAGGGCAATTTGCCTCATTCTG
NRPS10F	Pc21g15480	CTTGGTGGATGCAGCGAAGG
NRPS10R	Pc21g15480	CTGTGAGAGAGGCTCTTGAGTA
NRPS11F	Pc22g20400	TTCGCGAACATCCGAAGAAGC
NRPS11R	Pc22g20400	TCGGGCGAAGACACTGTTCA
pcbAB F	Pc21g21390	CACTTGACGTTGCGCACCGGTC
pcbAB R	Pc21g21390	CTGGTGGGTGAGAACCTGACAG
12.10 F	Pc21g05010	GATGCTTTCTTGAATAGCGGATCCACG
12.10R	Pc21g05010	GAACTTTGCGTTGATCGACTATTTCCACG
12.20 F	Pc21g05020	CTTAACATCTCCGATTCCCTACTCGG
12.20R	Pc21g05020	CAACCAATCCTGCGATCCATCCTCC
12.30 F	Pc21g05030	GGATAGCCAGAGAGTCAGAGAGACC
12.30R	Pc21g05030	GCGGACGGCAAGACTCAAGGCGCCTCC
12.40 F	Pc21g05040	GGAGAAAGCACGGCAATCGATAGTGG
12.40R	Pc21g05040	CCTATTCCTGTGCATGATTCTCGGCG
12.50 F	Pc21g05050	GAAGCGTGGATAGAGACCGAAGAGAAC
12.50R	Pc21g05050	GGAGCCAGACTCCGGAAAGGATACTG
12.60 F	Pc21g05060	GATAGTGAGTACAAATGCGCCTGGACC
12.60R	Pc21g05060	CGTTCAATACCGGAAATGGCTAGATTCG
12.90 F	Pc21g05090	CGTTAACTAATGACGCCACCTGTTGC
12.90R	Pc21g05090	GGAAAATAGTATCCCCAGCGATTGGC
12.100 F	Pc21g05100	CATCAGCACCGAGGTCTTCATTGTCG
12.100R	Pc21g05100	GCAACGCAATAGATGGTCAATGCCAG
12.110 F	Pc21g05110	CTGCAGCACTTCAGCATGGATGAAACC
12.110R	Pc21g05110	TCGTTGTGAGACTTGGATGCTCGGACG
12.120 F	Pc21g05120	CCTGCTTCTTAATCTTGCCCTGGC
12.120R	Pc21g05120	CCAAGCCGATGCCAAGAAGGAAGAG
12.130 F	Pc21g05130	CCTAAGTCCTTGATCAGAGCCTGG
12.130R	Pc21g05130	GTTGAGGTCGATACCCGTAAGTCATCC

## Additional files

Additional file 1: Table S1. The list of coding mutations acquired by the strain lineage of *P. chrysogenum.* (XLSX 52 kb) Additional file 2: Table S2. The list of COG/KOG/BRITE functional categories and distribution of the mutated nucleotides. (XLSX 354 kb)

## Abbreviations

6-APA: 6-aminopenicillanic acid
BLAST: Basic local alignment search tool
CSI: Classical strain improvement
COG: Clusters of orthologous groups
GASVPro: Geometric Analysis of Structural Variants
gDNA: Genomic DNA
HR: Highly reductive
KEGG: The Kyoto encyclopedia of genes and genomes database
KOG: euKaryotic Ortholog Groups
LC-MS: Liquid chromatography-Mass spectrometry
NR: Non reductive
NRPS: Nonribosomal peptide synthetase
PKS: Polyketide synthase
SNP: Single nucleotide polymorphism
q-PCR: Real-time quantitative PCR
